# Improving availability, promotion and purchase of fruit and vegetable and non sugar-sweetened drink products at community sporting clubs: a randomised trial

**DOI:** 10.1186/s12966-015-0193-5

**Published:** 2015-03-10

**Authors:** Luke Wolfenden, Melanie Kingsland, Bosco C Rowland, Pennie Dodds, Karen Gillham, Sze Lin Yoong, Maree Sidey, John Wiggers

**Affiliations:** School of Medicine and Public Health, The University of Newcastle, Callaghan, NSW 2308 Australia; Hunter New England Population Health, Wallsend, NSW 2287 Australia; Deakin University, Burwood, VIC 3125 Australia; Australian Drug Foundation, Melbourne, VIC 3000 Australia

**Keywords:** Sport, Prevention, Obesity, Nutrition, Diet, Adult

## Abstract

**Background:**

Amateur sporting clubs represent an attractive setting for health promotion. This study assesses the impact of a multi-component intervention on the availability, promotion and purchase of fruit and vegetable and non sugar -sweetened drink products from community sporting club canteens. We also assessed the impact the intervention on sporting club revenue from the sale of food and beverages.

**Method:**

A repeat cross-sectional, parallel group, cluster randomized controlled trial was undertaken with amateur community football clubs in New South Wales, Australia. The intervention was conducted over 2.5 winter sporting seasons and sought to improve the availability and promotion of fruit and vegetables and non sugar-sweetened drinks in sporting club canteens. Trial outcomes were assessed via telephone surveys of sporting club representatives and members.

**Results:**

Eighty five sporting clubs and 1143 club members participated in the study. Relative to the control group, at follow-up, clubs allocated to the intervention were significantly more likely to have fruit and vegetable products available at the club canteen (OR = 5.13; 95% CI 1.70-15.38), were more likely to promote fruit and vegetable selection using reduced pricing and meal deals (OR = 34.48; 95% CI 4.18-250.00) and members of intervention clubs were more likely to report purchase of fruit and vegetable (OR = 2.58 95% CI; 1.08-6.18) and non sugar -sweetened drink (OR = 1.56; 95% CI 1.09-2.25) products. There was no significant difference between groups in the annual club revenue from food and non-alcoholic beverage sales.

**Conclusion:**

The findings demonstrate that the intervention can improve the nutrition environment of sporting clubs and the purchasing behaviour of members.

**Trial registration:**

Australian New Zealand Clinical Trials Registry: ACTRN12609000224224.

## Introduction

Improving public health nutrition represents one of the most promising strategies to averting premature morbidity and mortality from chronic conditions including obesity, cancer and cardiovascular disease [1]. Inadequate fruit and vegetable intake accounts for 5.8% of disability adjusted life years globally [2]. Similarly, increasing the consumption of non sugar-sweetened beverages, in particular water, on its own [3] or accompanied by a reduction in sugar-sweetened beverages [4], is associated with a lower risk of obesity and reduced total energy intake [5,6].

Consistent with socio-ecological perspectives on health [7], the World Health Organization [8] recommends a settings-approach to improve public health nutrition, whereby environments and organisations in which people frequent are modified so that they are more supportive of making healthy food choices. While health promotion interventions have been found to be effective in improving the nutrition environments of settings including the home [9], schools [10], or workplaces [11], maximising the public health benefits of setting based intervention requires implementation of nutrition initiatives across other community settings.

One setting that has been increasingly recognised as a promising environment to promote healthy eating is amateur community sport clubs [12]. In European countries [13], as well as countries such as Canada [14] and Australia [15], amateur sports participation is common. Sporting clubs therefore provide access to large numbers of adults each year. Furthermore, there is considerable scope to improve the nutrition promoting environment within sporting clubs. In Australia, for example, over 90% of sporting clubs sell sugar-sweetened drinks such as soft drink and sports drinks, just one-third sell fresh fruit or vegetables [16] and only 20% promote healthier foods to club members and spectators [17].

Despite the merits of promoting healthy eating at community sporting clubs a number of systematic reviews, for example, have found no controlled studies examining interventions to implement policies aimed at improving health behaviours in sporting organisations [18,19]. Similarly we have been unable to locate any such trials following an extensive search of the literature. The aim of this study was to assess the effect of a multi-component intervention on i) the availability of fruit and vegetable and non sugar-sweetened drink products at community sporting club canteens, ii) the promotion of fruit and vegetable and non sugar-sweetened drink products at community sporting club canteens and iii) sporting club member purchasing of fruit and vegetable and non sugar-sweetened drink products from community sporting club canteens. Through supporting clubs to increase the availability and promotion of non sugar-sweetened drinks and fruit and vegetable products it is hypothesised that sporting club members will be more likely to purchase such products. Given sporting club representatives cite concerns regarding the perishability, cost and lack of customer demand for healthy foods from canteens as a potential unintended adverse effect [16], the study also assessed the impact of the intervention on club income from the sale of foods and non-alcoholic beverages from the club canteen.

## Methods

### Design

A repeat cross-sectional, parallel group cluster randomized controlled trial was undertaken with football clubs (clusters) randomized to either control or intervention groups. The study was nested within a trial of an alcohol management accreditation intervention [20] undertaken in a region that included major cities and rural communities from the state of New South Wales, Australia. Healthy canteen strategies described in this manuscript were integrated into the first and second accreditation levels of the broader three-level alcohol management intervention, with progression of clubs from one level to the next being dependent on clubs implementing both the healthy canteen and alcohol strategies. In addition to working with the sporting club committee and bar staff on alcohol management, research staff (support staff) also assisted staff responsible for canteen operation to implement targeted healthy canteen availability and promotion strategies. The trial findings are reported consistent with the CONSORT statement [21].

### Participants and recruitment

#### Clubs

All community level, amateur clubs from four football codes (Australian Rules football, Rugby League, Rugby Union and soccer/association football (hereafter referred to as ‘football’) in the study area were eligible to participate if the club had over 40 members and sold food and alcohol (criteria of the broader randomised controlled trial). In Australia, amateur community football clubs are managed by voluntary club committees and officials. Players participate in weekly organised competition, typically at publicly owned venues during sporting seasons lasting approximately five months per year. A representative was nominated by each identified club to participate in eligibility screening and data collection on behalf of the club. Club representatives from all 328 identified clubs within the study area were interviewed by telephone and, if eligible, invited to participate.

#### Club members

Club members were eligible to participate in the study if they were at least 18 years of age, spoke English and were current members of the club (e.g. players, committee members, spectators/fans or coaches). A quasi-random selection process was used to recruit club members for both the baseline and post-intervention cross-sectional surveys, with study information sheets and consent forms distributed by club management to up to 30 members of the club with the most recent birthdays [22,23]. These members were telephoned by trained research personnel to formally confirm eligibility and consent to participate in the study.

### Random allocation and blinding

Following the completion of baseline data collection clubs were randomly allocated to intervention or control conditions using simple randomization in a 1:1 ratio, stratified by football code and geographic area (based on the postcode of the club). The randomization procedure was performed by a statistician who did not have access to club baseline data, and was not involved in intervention delivery or data collection. Allocation was undertaken using a random-number generator in Microsoft Excel. Research personnel involved in post-intervention data collection were blind to the group allocation of the participating football clubs.

### Intervention

Like most amateur sporting clubs in Australia, the sale, promotion and pricing of food and beverages through interventions club canteens was managed by volunteer staff [24]. Football sporting club canteens typically operate out of publically owned kiosks, are operational only during sporting events, and have limited food preparation, cooking, or food storage facilities. The intervention for this study sought to improve the availability and promotion of fruit and vegetables and non-sugar sweetened drinks in sporting club canteens, strategies which have been found to be effective in changing consumer purchasing behaviour [25].

The intervention was developed based on social-ecological models of health which suggest that individual, social, cultural and physical environmental factors that operate at multiple levels are key determinants of health behaviours [7]. There is little evidence regarding effective strategies to improve the selection of healthy foods in a community sporting club context. As such intervention strategies suggested to be effective in improving healthy food selection in analogous settings (e.g fast food restaurants) [26-31] were identified from the literature and selected to address physical, social, cultural and individual factors considered important in influencing club member’s canteen purchasing decisions by the research team. Such strategies were broadly characterised as those that targeted the availability and/or promotion (including pricing) of healthy food and beverages purchased. Specifically, over two and a half sporting club seasons, intervention clubs were supported by research staff to implement the following evidence based strategies:

1. **Availability of healthy food and beverage options**
  *Physical environment strategies*
  To improve the physical environment, clubs were to increase availability of fruit and vegetable and non-sugar sweetened beverages [27,29] by providing a total of six fruit and vegetable (such as fresh fruit, salads or salad sandwiches) and non sugar-sweetened drink (such as water and plain milk) products for sale at their club canteen. Further, clubs were required to ensure at least 75% of non-alcoholic drinks in the canteen fridge were non sugar-sweetened beverages and were positioned in the upper half of the fridge.
2. **Promotion of healthier food and beverage options**
  *Physical environment strategies*
  Promotional strategies to improve the physical environment included encouraging fruit and vegetables and non-sugar-sweetened drink purchase via meal deals (whereby fruit and vegetable products and water are packaged together at a reduce price), signage and posters to draw customers attention to such products [29]. Pricing strategies were also encouraged to ensure that fruit, vegetable and non-sugar sweetened drink products were priced competitively compared to similar less healthy products (such as pricing non-sugar sweetened beverages lower than sugar sweetened beverages) [26,27]. Furthermore, clubs were to ensure fruit and vegetable and non sugar-sweetened drink products were displayed within view of consumers at all times and prominently positioned either at eye level, upper half of the fridge or on the counter within the canteen [31].
  **Social and cultural strategies**
  To improve the social-cultural environment, the intervention targeted sporting club coaches and the club executive/committee as important sociocultural change agents within the clubs [30]. Specifically, coaches were asked to recommend all players drink water and consume fruit at half time and following competition games. Furthermore, club executive committee were required to develop a written food and nutrition policy that formally documented the club’s commitment to ongoing implementation of the healthy food intervention strategies as well as any additional related strategies the club may be undertaking such as limiting the involvement of the fast-food industry in club fundraising, sponsorship and advertising [28,32]. Policies were provided to members and staff and displayed in social rooms and reviewed annually.
  *Individual strategies*
  To improve individual club member awareness and attitudes regarding healthy foods and beverages clubs provided healthy food and drink guides/factsheets to parents/players each season. Clubs were also encouraged to display guides/factsheets in club social rooms and include them in player registration packs [33]. The fact sheets promoted the benefits of healthy foods and water consumption for sporting performance, and recovery as well as for good health generally.

Clubs could progressively work towards full implementation of the canteen strategies over the 2.5 sporting seasons or could implement all practices simultaneously. In keeping with the notion of developing a health promoting setting, clubs were also encouraged to adopt other strategies consistent with a healthier club canteen such as substitution of higher fat/energy products with lower fat/energy products (e.g. low fat pies and diet soft drink) and introduce other ‘healthier’ products (lower in energy, fat or sodium) for sale.

### Intervention implementation strategies

The following strategies were employed by the research team to support clubs implement the intervention:

1. *Human resources:* Each club was allocated a support officer to assist the club in implementing required healthy food and drink practices consistent with the criteria described above. Support officers attended a two-day training workshop prior to the delivery of the intervention followed by top-up training sessions and fortnightly supervision meetings with the research manager over the course of the intervention. Support was provided in the form of at least one face-to-face meeting with the club management committee, a face-to-face meeting with the canteen manager, and a follow-up telephone call and email to both club committee and canteen management staff over the 2.5 year intervention period. More frequent contact was provided (including face-to-face, email or phone contact) in instances where clubs requested support or where support staff noted clubs were experiencing difficulty implementing the canteen strategies and clubs were receptive to further contact. During contact with the club committee or canteen managers, support officers would assess current practice/progress toward strategy implementation, set strategy implementation goals, assist action planning and undertake problem solving.
2. *Recognition and Reward:* Implementation of required healthy food and drink practices were recognised and rewarded through an accreditation framework. Accreditation was achieved when clubs made available and promoted fruit, vegetable and non sugar-sweetened beverage products consistent with that required by the intervention. Incentives such as a certificate of accreditation and merchandise (e.g. counter mats, posters) were provided to clubs when accreditation standards were met.
3. *Resources*: All clubs received a comprehensive hardcopy resource kit that included an implementation manual as well as sample polices, pricing guides, recipes, templates for promotional signs and nutrition and sport information. Electronic versions of these resources were also provided as well as regular newsletter updates throughout the intervention period.
4. *Workforce development:* All clubs were provided with access to online training in nutrition and safe food handling. At least one person from each club, including the person responsible for stocking the canteen, was required to undertake the training. The online training included interactive quizzes and covered dietary guidelines, healthy food and drink options for sports clubs, and safe storage, preparation and handling of food.
5. *Audit and Feedback*: Observational performance audits of intervention group club canteens were conducted during football matches at least once per season during the intervention period to assess and feedback to clubs the implementation of intervention strategies by canteen staff.

### Control group

During the intervention period, control club management received printed resources on topics unrelated to the trial outcomes (such as illicit drug use), to distribute to club members. The intervention clubs also received these resources.

### Data collection procedures and measures

Baseline data were collected June-August 2009 and post-intervention data July-October 2012 via scripted telephone survey. All survey scripts were pilot-tested prior to use.

#### Club characteristics

Club representatives were asked to provide information regarding the following characteristics of their club: size (number of players and members), football code and postcode of sporting club venue.

#### Fruit and vegetable and non sugar-sweetened drink availability, promotion and purchase

At baseline and post-intervention, computer-assisted telephone interview (CATI) [34] surveys were conducted with club representatives to assess the availability of healthy food and drink products for purchase from club canteens as well as pricing and promotional strategies for these products. Specifically to assess availability, club representatives were asked to report if their club canteen sold fruit and vegetable products including fresh fruit, vegetables, salad or salad sandwiches; and if they sold the following non sugar-sweetened beverages: water, plain milk, diet soft drink (soda). To assess promotional strategies, club representatives were asked to report: if they used promotional strategies such as ‘meal deals’ or price discounting of fruit and vegetable products to encourage their consumption; whether coaches at their club recommend players consume fruit and water; and the proportion of refrigerator space dedicated to healthy drink products, which was estimated through a process of counting shelves.

Baseline and post-intervention CATI surveys were also conducted with club members from intervention and control groups to assess their purchase of healthy food (fruit and vegetable products) and unsweetened drink products (water, plain milk or diet soft drink) at the sports club. Specifically club members were asked “What foods do you usually purchase for your own consumption from the clubs canteen or shop” and “What drinks do you usually purchase for your own consumption from the clubs canteen or shop?”

#### Club revenue

Computer-assisted telephone surveys [34] were conducted with club representatives, who were asked to estimate their club’s approximate total income from food and non-alcoholic drinks over the past year. To improve validity of self-reported club revenue, a time to complete the telephone survey was arranged with participants to allow them to consult other club members or obtain financial records in preparation for the interview.

### Statistical analysis

Descriptive statistics were used to describe club and club member characteristics. Club postcode was used to classify clubs as being in a ‘major city’ or ‘inner/outer regional’ area based on the Australian Standard Geographical Classification [35]. Fisher’s Exact and Wilcoxon tests were used to assess potential bias due to differences in clubs lost to follow-up across the two treatment groups. Fisher’s Exact and Wilcoxon tests were also used to test the assumption of blinding of research staff.

For the trial outcomes, intention-to-treat analyses examining between-group differences over time (controlling for baseline) were undertaken using logistic regression for the dichotomous outcomes and linear regression for continuous outcomes. Availability, promotion and revenue outcomes were analysed at club level. Equivalent non-parametric tests (Wilcoxon Rank Sum test) were conducted to confirm findings for revenue measures as data were not normally distributed. Analyses of data to assess club member purchasing of fruit, vegetable and non sugar-sweetened beverages was assessed at an individual club member level, accounting for clustering within clubs using regression models within a Generalised Estimating Equations framework. Between group differences were assessed through an interaction of group and time variables in the model. The α-value for significance testing was 0.05. A sensitivity analysis was performed to test for any bias due to missing data, in which missing post-intervention data for clubs was imputed by carrying forward data collected for the club at baseline. The results of sensitivity analyses are reported when they differed from those of the main analyses (p > or <0.05). SAS (version 9.2) was used for all statistical analyses.

### Sample size and power calculations

Unpublished data previously collected by the research team indicated that the prevalence of healthy food (fruit and vegetable product) purchase at sporting clubs was approximately 7%. Based on this figure and allowing for an intra-class correlation of 0.5, it was determined that 35 clubs per experimental group (with 19 members per club) would provide the study with 80% power to detect a 17% difference in the prevalence of healthy food purchase. It was also calculated that 35 clubs per group would provide the study with 80% power to detect a difference of 33% in the prevalence of healthy food (fruit and vegetable product) availability, based on a baseline prevalence of approximately 40% (unpublished data).

## Results

**Figure 1:**
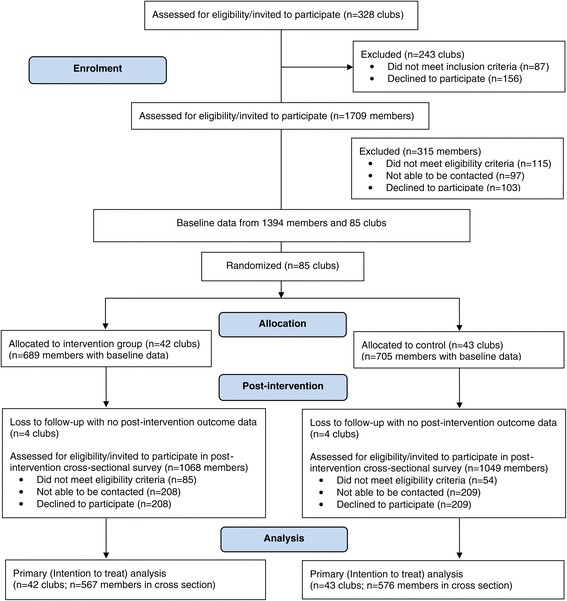
**Consort flowchart describing progress of clubs and members through the trial.**

Three hundred and twenty eight potentially eligible clubs were identified in the study area, of which 241 were determined to be eligible following screening and were invited to participate in the trial (see Figure 1). Of these, 85 (36%) consented to participate. Consenting clubs did not differ significantly from non-consenting clubs in terms of football code (χ2 = 6.68 df = 3; p = 0.08) or location (major city; inner regional) (χ2 = 0.20 df = 1; p =0.66). The 85 clubs that consented to participate were randomly allocated to control (N = 43) and intervention (N = 42) conditions.

Baseline characteristics of participating club members are also show in Table 1. Across both intervention and control groups, participating clubs had an average of over 250 members at baseline and the majority of club venues were located in major city areas. Club characteristics were similar across control and intervention groups. No significant differences existed between the percentage of clubs in the intervention and control groups that were lost to follow-up (10% vs 9%; Figure 1).

**Table 1:** **Baseline characteristics of participating football clubs and club members**

**Characteristic**		**Control clubs (43 clubs; 689 club members)**	**Intervention clubs (42 clubs; 705 club members)**
Clubs			
Football code	Australian Rules	15.6%	16.7%
	Rugby League	33.3%	31.0%
	Soccer/association football	24.4%	19.0%
	Rugby Union	26.7%	33.3%
Geographical region	Major city	80.0%	83.3%
	Inner/outer regional	20.0%	16.7%
Size	Mean number of players (SD)	272 (235)	259 (360)
Role	Players	47.0%	60.1%
	Spectator/other members	18.3%	13.9%
	Club committee members	18.3%	12.1%
	Coaches/umpires/referees	16.5%	13.9%
Members			
Age of members	Mean (SD)	32.7 (12.0)	36.0 (11.9)
Gender	Male	87.0%	77.4%
Education	University Educated	23.2%	21.0%
Income	More than AU$52 000	48.0%	49.3%

See Figure 1 for club member participation in the trial. Baseline data were collected from 1394 club members. The post-intervention cross-sectional survey included 567 members of intervention group clubs and 576 members of control group clubs. As shown in Table 1, both intervention and control groups had approximately one-fifth of participants with a university-level education at baseline and just under half with an income above AU$52,000. Players were the largest group of survey participants (intervention: 60% and control:47%).

### Fruit and vegetable and non sugar-sweetened drink availability

Post-intervention, clubs receiving the intervention reported a significant increase in the availability of fruit and vegetable products (OR = 5.13; 95% CI 1.70-15.38) at club canteens compared to control group clubs (Table 2). There were no statistically significant differences between groups in the reported availability of non sugar-sweetened drink products.

**Table 2:** **Healthy food and drink availability and promotion at baseline and post-intervention by group**

	**Baseline**		**Post-intervention**			
	**Control clubs**	**Intervention clubs**	**Control clubs**	**Intervention clubs**	**OR**	**p-value**
	**n (%)**	**n (%)**	**n (%)**	**n (%)**	**(95% CI)** ^**a**^	
Availability of fruit and vegetable products at club canteen	15 (35%)	19 (45%)	19 (49%)	31 (82%)	5.13 (1.70-15.38)	0.006
Availability of non-sugar sweetened drinks at club canteen	43 (96%)	41 (100%)	28 (93%)	31 (100%)	0.38 (0-3.22)	0.459
Fruit and vegetables promoted via meal deals and reduced pricing	2 (4.7%)	3 (7.1%)	1 (2.6%)	18 (47.0%)	34.48 (4.18-250.00)	<0.001
Coaches recommend fruit or water	35 (81.0%)	37 (88.0%)	36 (92.0%)	34 (89.0%)	0.69 (0.14-3.40)	0.955
% of drink space in canteen fridge occupied by water and plain milk (mean and SD)	21.72 (13.15)	17.13 (6.68)	19.63 (8.06)	21.83 (15.68)	-	0.665

^a^Odds ratio for the intervention group compared to the control group at post-intervention.

### Fruit and vegetable and non sugar-sweetened drink promotion

As shown in Table 2, the proportion of intervention clubs offering meal deals and reduced pricing to promote fruit and vegetable products significantly increased following the intervention (OR = 34.48; 95% CI 4.18-250.00) compared with control clubs. There were no significant differences between groups in use of other promotional strategies.

### Fruit and vegetable and non sugar-sweetened drink purchase

Also shown in Table 3, the proportion of intervention club members reporting purchasing fruit and vegetable products increased significantly relative to members of control clubs (OR = 2.58 95% CI; 1.08-6.18). Similarly, reported purchase of non sugar-sweetened drinks increased significantly amongst members of intervention clubs compared to members of control clubs (OR = 1.56; 95% CI 1.09-2.25).

**Table 3:** **Healthy food and drink purchase at baseline and post-intervention by group**

	**Baseline**		**Post-intervention**			
	**Control club members**	**Intervention club members**	**Control club members**	**Intervention club members**	**OR (95% CI)** ^**a**^	**p-value**
	**n (%)**	**n (%)**	**n (%)**	**n (%)**		
Usual purchase of fruit and vegetable products by members	56 (7.9%)	49 (7.1%)	52 (9.0%)	105 (18.5%)	2.58 (1.08-6.18)^a^	0.033^b^
Usual purchase of non sugar-sweetened drinks by members	273 (38.7%)	245 (35.6%)	238 (41.3%)	278 (49.0%)	1.56 (1.09-2.25)^a^	0.015^b^

^a^Relative odds ratio for the interaction term.

^b^Adjusted for clustering at club level.

### Club revenue

At baseline, intervention group clubs reported a mean total annual revenue from food and non-alcohol drinks of AU$32,015 (SD: AU$35,058) and control group clubs a mean total annual revenue of AU$23,000 (SD: AU$25,748). Adjusting for baseline, there was no significant difference in club annual revenue between intervention AU$29,669 (SD: AU$31,205) and control group clubs AU$26,529 (SD: AU$33,465) following the intervention (*p =* 0.910).

## Discussion

To date, community sporting club canteens have been mainly characterised by the sale and promotion of energy dense, nutrient poor foods and beverages [16]. The findings of this study demonstrate that the intervention can improve the availability and promotion of fruit and vegetable products and non sugar-sweetened beverages and increase the purchase of these products by sporting club members. As the first randomised controlled trial of a nutrition intervention in this setting, the trial makes an important and novel contribution for researchers, policy makers and sporting organisations interested in improving public health nutrition.

At follow-up, 82% of intervention clubs provided fruit and vegetable products for sale, a marked improvement relative to control clubs (45%). This effect size is comparatively larger than that reported for initiatives to improve fruit and vegetable availability in schools, where availability of these products has typically increased between 0-20% [10,36-39]. These findings support previous research indicating that sporting clubs may be amenable to becoming more health promoting environments [16]. Furthermore, the findings suggest that the intervention enabled clubs to overcome previously reported barriers to introducing fruit and vegetables products such as a perceived lack of consumer demand and concerns regarding profitability [16]. For example, consistent with research in the school canteen setting [36,40], this study found fruit and vegetable and non sugar-sweetened drinks were commonly purchased (by 19% and 49% of club members respectively) and no evidence that the introduction of healthy products such as fruit and vegetables reduced club income from non-alcoholic food and beverage sales.

The increase in purchasing of non sugar-sweetened drinks by members of intervention clubs was surprising given that the availability of these products in club canteens was high (>90%) at baseline, and that there was no significant improvement in the amount of fridge space allocated to these products. Potentially, the increase in purchasing of non sugar-sweetened drinks reflects significant increases in the use of ‘meal deal’ promotions by intervention clubs (47% of intervention clubs conducted such pricing promotions post-intervention compared with 3% of control clubs), which anecdotally often include the discounting of a healthy food and drink combination (e.g. a salad sandwich and a bottle of water). If so, the findings are consistent with previous research indicating that price is a key driver of consumer purchases and a reduction in prices is a powerful tool in encouraging healthy choices [40,41].

The primary limitation of the trial was its reliance on self-report assessment of trial outcomes. Direct observation or sales data would have provided more valid estimate of product availability, promotion and purchase. The internal validity of the trial would have also been strengthened had the survey items in this study been validated. The trial was nested within a broader randomised trial assessing the impact of an intervention to improve alcohol management at community sporting clubs. As such, there is potential that the simultaneous implementation of interventions in these clubs may have interacted in a way that impacted on the trial findings. For some clubs, for example, having to address alcohol management practices may have reduced the available resources within the club to implement healthy canteen strategies. If this was the case, support targeting canteen strategy implementation only may have yielded greater intervention effects than reported in this manuscript. Finally, the study recruited clubs from major football codes in Australia. The generalizability of the findings to other sporting clubs, or sporting clubs in other jurisdictions requires further study. Given the limitations of the study, future research should seek to confirm the trial findings using more objective measures of fruit and vegetable and sugar-sweetened drink availability and promotion, such as data collected through canteen audits, and on a more diverse sample of sporting clubs. Extending the research to sporting clubs with junior participants has also been identified as an important area for future research given the importance of childhood in establishing healthy dietary habits [42] and the promotion of unhealthy foods to children in this setting [43].

Despite its limitations, the trial identifies an effective model of improving the sporting club nutrition environment, as is recommended as part of a settings based approach to health promotion. Such findings should be of particular interest to health policy makers and sporting club organisations/management.

### Ethics approval

The study was approved by The University of Newcastle Human Research Ethics Committee (reference: H-2008-0432).

## Abbreviation

CATI: Computer-assisted telephone interview
